# Pilot implementation of an electronic patient-reported outcome measure for planning and monitoring participation-focused care in early intervention

**DOI:** 10.1186/s12911-020-01189-9

**Published:** 2020-08-24

**Authors:** E. C. Albrecht, V. C. Kaelin, B. L. Rigau, J. K. Dooling-Litfin, E. A. Scully, N. J. Murphy, B. M. McManus, M. A. Khetani, on behalf of the High Value Early Intervention Research Group, Jamie Bane, Haley Carle, Amy Jatsko, Amanda Pedrow, Laura Sciarcon

**Affiliations:** 1 Invest in Kids, 1775 Sherman Street, Suite 1445, Denver, CO 80203 USA; 2 Rehabilitation Sciences, College of Applied Health Sciences, University of Illinois at Chicago, Chicago, IL 60612 USA; 3 Department of Occupational Therapy, College of Applied Health Sciences, University of Illinois at Chicago, 1919 West Taylor Street, Room 316A, Chicago, IL 60612-7250 USA; 4 Rocky Mountain Human Services, 9900 E Iliff Ave, Denver, CO 8023 USA; 5 Health Systems, Management, and Policy, Colorado School of Public Health, 13001 E. 17th Place, Mail Stop B119, Aurora, CO 80045 USA; 6 CanChild Centre for Childhood Disability Research, McMaster University, 1280 Main Street West, Hamilton, Ontario L8S 4L8 Canada

**Keywords:** Family-centered care, Young children, Participation, Online, Care planning, Collaborative goal-setting, Early intervention

## Abstract

**Background:**

Family-centered care is a valued approach to improving child and family outcomes in early intervention (EI), yet there is need to implement interventions that support information exchange for shared decision-making when planning and monitoring EI care. This study aims at estimating the feasibility, acceptability, and value of implementing the Young Children’s Participation and Environment Measure (YC-PEM), a valid electronic patient-reported outcome (e-PRO) that is designed to support family engagement when planning care and monitoring outcomes of care.

**Methods:**

Data were gathered from caregivers (*N* = 139) that were enrolled in a Phase 1 trial of the YC-PEM e-PRO as implemented within 1 month of their child’s next EI evaluation of progress. YC-PEM e-PRO feasibility was estimated according to enrollment and completion rates, and mean completion time. Chi-square tests were used to examine parent perceptions of YC-PEM e-PRO acceptability by caregiver education and family income. Caregiver feedback via open-ended responses were content coded to inform intervention and protocol optimizations. YC-PEM e-PRO value was estimated via composite and item-level scores to capture the extent of participation difficulty in home and community activities, and common areas of need regarding caregivers desired change in their child’s participation.

**Results:**

Feasibility of implementing the YC-PEM e-PRO in routine EI care was mixed, as evidenced by low enrollment rates (21.0–29.2%), a high completion rate (85.3%), and limited missing data (80.6% of completed cases contained no missing data). More than half of the participants reported that the completion of the YC-PEM e-PRO was at least somewhat helpful, regardless of family income or caregiver education, providing support for its acceptability. As for its value, the YC-PEM e-PRO results were viewed by 64% of caregivers, whose desire for change most often pertained to the child’s participation in non-discretionary activities at home and structured activities in the community.

**Conclusions:**

Results may support the implementation of YC-PEM e-PRO as a feasible, acceptable, and valued option for engaging families in planning the child’s EI care. Results also inform select intervention and protocol optimizations prior to undertaking a multi-site pragmatic trial of its effectiveness on family engagement and shared decision-making within an EI clinical workflow.

**Trial registration:**

Trial number: NCT03904797. Trial registered at Clinicaltrials.gov. Registered 22 March 2019. Retrospectively registered.

## Background

Early intervention (EI) is a federally funded, state administered service system through Part C of the Individuals with Disabilities Education Act. EI provides access to rehabilitation and developmental services for approximately 2–3% of infants and young children with developmental needs nationally [1]. Children are deemed eligible for EI services if they have a diagnosed disability (e.g., cerebral palsy), developmental delay based on standardized test scores (e.g., fine motor delay), or are at risk for developmental delay (e.g., very low birth weight). EI services are delivered within the child’s natural learning environment (e.g., home, community) [2–4], to improve 1) family caregiving capacity, including their ability to help their child to participate in activities that foster the child’s skill development; and 2) the child’s cognitive, social, and adaptive functioning. Family-centered care (FCC) is a federally mandated approach to delivering EI care and involves providers partnering with families to design and monitor care that is responsive to family priorities for their child’s participation in valued activities [3].

Despite its importance, EI providers struggle to implement family-centered care in their clinical workflows [5–7]. Prior studies suggest that approximately one-third of enrolled families nationally report dissatisfaction with the family-centeredness of their child’s EI care [8]. Family disengagement is linked to less robust implementation of intervention strategies and poorer child outcomes [9].

One key opportunity to implement FCC within an EI clinical workflow is when planning care. However, designing family-centered and participation-focused care often relies on a standard face-to-face approach and takes considerable provider and family resources to complete [10]. While caregivers have expertise on the activities that are problematic for their child [6], current literature shows that they may lack self-efficacy or sufficient resources to identify and communicate their concerns to members of the child’s EI team [11, 12].

As EI programs transition to electronic data capture for purposes of accountability and quality improvement [13, 14], the implementation of electronic assessment options may be a scalable strategy that EI programs can use to give families options for providing input about their child’s EI care [15–17]. The Young Children’s Participation and Environment Measure (YC-PEM) is an electronic patient-reported outcome (e-PRO) measure that is designed to support FCC by giving caregivers a valid and reliable way to communicate about their child’s current participation and areas of participation need, while also allowing EI programs to aggregate these data to examine trends in this patient-important outcome over time [17–21].

To assess its clinical utility prior to full scale deployment within an EI system of care, the YC-PEM e-PRO was implemented with EI programs that have electronic data capture. It was first implemented with a small university-affiliated EI program (annual enrollment: 80 families) to examine its feasibility, value, and acceptability. Feasibility was mixed when the YC-PEM e-PRO was introduced by EI providers during EI service visits. While less than half of eligible families (i.e., 44%) opted to complete the YC-PEM e-PRO over a 2.5 month period, there were no missing e-PRO data among those who completed it [22]. The YC-PEM e-PRO was found to be valuable in detecting the extent to which caregivers wanted their child’s participation to change, and could be used to show significant associations between EI service intensity and the child’s level of participation in valued home activities [22]. In providing feedback on its acceptability, EI providers suggested that the YC-PEM e-PRO be tested when it is implemented as part of the child’s annual evaluation of progress, rather than the EI service visit, because EI service coordinators routinely assess for participation need when assessing the child’s progress on an annual basis [22].

Since university-affiliated EI programs have stronger provider capacity for research engagement, a logical next step for assessing the scalability of implementing the YC-PEM e-PRO assessment across diverse EI programs is to establish its feasibility, value, and acceptability when deployed in larger, non-university affiliated EI programs. In non-university affiliated EI programs, EI providers’ lack of routine involvement in research may contribute to disengagement with testing YC-PEM e-PRO implementation, thereby making it a key setting to understand implementation [16]. There is need to establish YC-PEM feasibility when implemented into routine annual evaluations of the child’s progress, YC-PEM value for understanding common areas of family need to address in an individualized family service plan, and YC-PEM acceptability from the parent perspective [23]. This will help to 1) determine whether the implementation of the YC-PEM e-PRO is practical [24], 2) identify further assessment and protocol modifications needed prior to future implementation efforts, and 3) assure individual site preparedness for implementation [24] and further testing of the intervention [16].

The purpose of this study is to assess the feasibility (aim 1; i.e., enrollment rates, completion rate, completion time), acceptability (aim 2; i.e., caregiver perspective on usefulness of YC-PEM), and value (aim 3; i.e., percent of caregivers accessing online report, percent of caregivers reporting desire for change in their child’s participation) of implementing the YC-PEM e-PRO as an evidence-based option to customize family assessment in an EI workflow. Study results will inform YC-PEM e-PRO optimizations and protocol modifications prior to undertaking a multi-site pragmatic trial.

## Methods

### Participants and setting

This is a single-arm, non-randomized pilot implementation trial [25]. Multi-institutional ethics approval was obtained prior to recruitment and data collection (March 2017–August 2018) (University of Illinois at Chicago, #2016–0139). Participants were family caregivers (*n* = 149), the end users of the YC-PEM e-PRO, and were recruited from a large, urban, and non-university affiliated EI program. This EI program was reported to serve nearly 1000 families of children 0–3 years of age annually and has reported less provider familiarity with research engagement [16]. During the 18-month data collection period, a total of 776 caregivers were eligible to be invited into this study. These families were therefore approached by EI staff 1 month prior to the child’s annual evaluation of progress. Each caregiver confirmed his or her eligibility online by verifying that they were at least 18 years old; could read, write, and speak English or Spanish; had internet access; and had a child between 0 and 3 years old who had received EI for at least 3 months. For this study, we analyzed a subset of data on families who completed the English version of the YC-PEM e-PRO (*n* = 139), because the English but not the Spanish version has been previously validated in terms of its psychometric properties [17–21].

### YC-PEM e-PRO intervention

The YC-PEM e-PRO is an electronic health tool that caregivers can use to evaluate their young child’s participation in valued activities within the home (e.g., mealtime), daycare/pre-school (e.g., classroom learning), and community settings (e.g., community events). Completion time for the entire YC-PEM e-PRO is 30–40 min [26]. When completing the YC-PEM e-PRO, caregivers answer questions about how often their child participates in activities (7-point scale, from never to once or more each day), their child’s level of involvement in those activities (5-point scale, from not very involved to very involved), and their desire for their child’s participation to change (yes, no). After evaluating their child’s participation, caregivers are asked about the impact of various environmental features (e.g., physical layout of the home) and resources (e.g., information and supplies) on their child’s participation in activities within a setting. Details of the YC-PEM e-PRO have been described elsewhere [17–21]. For this study, caregivers were instructed to complete two of three sections of the YC-PEM e-PRO (i.e., home and community) within a month, prior to the child’s annual evaluation of progress. The decision to administer the home and community sections was made in consultation with the EI program, and based on low and variable daycare/preschool enrollment rates among their enrolled families. This approach to decision-making about trial design is congruent with a community-engaged research approach [16].

### Measures

Measure selection was informed by the Family of Participation-Related Constructs (fPRC) [19, 27, 28] and a systematic review of salient child and family status and process factors that are associated with children’s participation [29]. The fPRC is a contemporary framework that defines participation as the child’s attendance and level of involvement in activities, as influenced by intrinsic and extrinsic factors such as a child’s environment [27].

#### Child and family characteristics and early intervention service use

Caregivers who expressed interest, and confirmed their eligibility online to participate in the study were directed to an online demographic questionnaire (*n* = 163) to report on their level of education, age, marital status, race, relationship to child, and family income. Additionally, caregivers reported on their child’s social and clinical characteristics, including the child’s age, race, ethnicity, sex, and developmental condition type (diagnosis versus developmental delay).

EI service use was captured via record abstraction and reported out according to amount (hours), duration (months), intensity (hours per month), and type. EI service amount was estimated as the total number of hours of EI services, as well as the total number of hours per each of the following four core EI services: physical therapy (PT), occupational therapy (OT), speech therapy (ST), and developmental intervention (DI). Service duration was calculated by subtracting the date of EI entry (i.e., the child’s date of EI eligibility evaluation) from the date of study enrollment, as reported in months. Service intensity (hours per month) was then derived by dividing total service amount (hours) by service duration (months), to yield an estimate of total hours per month of EI services. Receipt of each of the four core EI services were also included as number of EI services received (one, two, three or more).

#### YC-PEM e-PRO feasibility

The feasibility of implementing the YC-PEM e-PRO within an EI workflow was assessed via web analytic data on study enrollment rates (number of families who were approached by EI staff, expressed interest, and confirmed their eligibility online), completion rate (number of families who completed the YC-PEM e-PRO online), and mean completion time (minutes).

#### YC-PEM e-PRO acceptability

Following completion of the YC-PEM e-PRO online, caregivers were invited to share their perceptions of how useful YC-PEM e-PRO was for planning care by their responses to the question, “Did you find this survey helpful for understanding your child’s participation in the home and community? Why or why not?”

#### YC-PEM e-PRO value

For this study, we estimated value in two ways. First, we used web analytic data to estimate the percentage of families who accessed their child’s online summary report following e-PRO completion. Second, when completing the YC-PEM e-PRO, caregivers identified whether or not they desired a change (yes, no) in their child’s current participation within specific activities at home (13 activities) and/or the community (11 activities). These data highlight areas of parent engagement with EI care as well as caregiver dissatisfaction with the child’s participation. Therefore, these data may provide key insight into areas of family need. Since the purpose of family assessment is to solicit information about family need to inform intervention priorities, these YC-PEM e-PRO data are presented to show their value for EI family assessment.

For this study, a *percent desire change* score was calculated for each item and as a mean score. To calculate the percent desire change score per item, the number of ‘yes, change desired’ responses were summed up, divided by the total number of responses, and multiplying by 100. These item scores afford for detailed description of common areas of participation need across activities within each setting. The mean percent desire change composite score was derived for each setting (home, community), by taking the average across individual setting scores, which were calculated by summing the number of ‘yes’ responses across items, dividing by the number of items, and multiplying by 100. The YC-PEM e-PRO internal consistency reliability estimates were considered good for data obtained in this study (ɑ = .80 for home desire change, ɑ = .84 for community desire change) [30].

### Data collection

Twenty-two EI service coordinators participated in a 90-min video-conference session to learn about project purpose, provide feedback on recruitment processes and materials, and learn how to enroll participants via the project website. Service coordinators who completed this session used a script and flyer to then recruit families during the scheduling of their child’s annual review of EI progress. The recruitment protocol was modified in response to low enrollment [16], such that a designated EI staff member was paired with research staff to recruit participants. Further details about protocol modifications are published elsewhere [16].

Eligible and interested caregivers visited the project website to create an account, confirmed study eligibility, provided informed consent and HIPAA authorization for abstracting select EI service use data, and completed a demographic questionnaire and the YC-PEM e-PRO. Caregivers received immediate access to an online report summarizing their e-PRO responses to share with their child’s EI team and were mailed $10.00 gift cards after e-PRO completion.

### Data analysis

SPSS 24.0 was used to perform all analyses for this study. Descriptive statistics were used to summarize sample characteristics and EI service utilization according to service dosage (amount, duration, intensity) and number of core EI services. For continuous variables, sample means, medians, and standard deviations were calculated. Due to skewed distribution, inter-quartile ranges were used to describe EI service utilization. Sample proportions were calculated for categorical variables.

For YC-PEM e-PRO feasibility (aim 1), enrollment and completion rates, as well as mean completion time were estimated for the 139 families who completed the English YC-PEM e-PRO. The success of feasibility was determined as e-PRO enrollment and completion rates of 50% or higher based on family assessment completion rate within usual care (personal conversation with E.A. Scully, March 1, 2019). Therefore, the criterion for determining whether the YC-PEM e-PRO is feasible is *n* = 388 participants (i.e., 50% of the 776 eligible participants), as has been done in previous studies [22].

For YC-PEM e-PRO acceptability (aim 2), caregiver feedback via open-ended responses were coded using two approaches. In the first coding approach, responses were sorted into a one of three categories (helpful; somewhat helpful; not helpful) to create a new variable that captured the extent to which caregivers perceived the YC-PEM e-PRO to be useful for planning EI care. Given the previously reported positive association between participation, and family education and income [31], chi-square tests were used to examine perceptions of YC-PEM e-PRO acceptability by caregiver education (high school or some college; college degree; graduate training) and family income ($0–$50,000; $50,001–$100,000; > $100,001) [32]. The criterion we used to evaluate whether acceptability was associated with caregiver’s level of education or family income was *p* < .05.

In the second coding approach, responses underwent inductive content analysis [33], whereby data were grouped into categories to identify supportive and problematic aspects of the user experience. Data were summed for each category, to help interpret the salience of each aspect described. Supportive aspects were expected to inform optimizations to the recruitment protocol, whereas aspects of concern were intended to inform optimizations to the YC-PEM e-PRO prior to further testing. Study staff independently coded each open-ended response and met to resolve coding discrepancies through discussion, resulting in the development of a new category to best fit the data.

For YC-PEM e-PRO value (aim 3), the percentage of participants who viewed a summary of their e-PRO responses via an online report was estimated. In addition, mean percent desire change scores were calculated for each setting (home, community) to identify the extent of participation difficulty in each context, and item scores were rank ordered to identify common areas of home and community participation need.

## Results

### Sample characteristics

As shown in Table 1, more than half of the children included in this study were between 24 and 35 months old (54.0%), male (51.1%), and had a developmental delay (no diagnosis) (72.7%). On average, children had received EI services for 13.87 months. Most of the children received multiple EI services, with the most common services being ST (69.1%) and PT (52.5%).

**Table 1:** Child and family social characteristics and early intervention service use at study enrollment

Characteristic	*N* = 139 (%)		Mean (SD)
Child Sex, Male^a^	71 (51.1)	Service Duration (months)^a^	13.87 (5.70) **n (%)**
Insurance Type, Public Insurance^a^	39 (28.1)	Type of EI Services Received^a^	
Had a Diagnosis	38 (27.3)	PT	73 (52.5)
Child Age (months)		OT	44 (31.7)
12 to 24	64 (46.0)	ST	96 (69.1)
over 24	75 (54.0)	DI	51 (36.7)
Child Race^ab^		Number of EI Services Received^a^	
White	104 (74.8)	1	37 (26.6)
Black	9 (6.5)	2	57 (41.0)
Asian	2 (1.4)	3 or more	36 (25.9)
American Indian/Alaska Native	2 (1.4)		**Median [IQR]**
Native Hawaiian/ Other Pacific Islander	1 (.7)	Total Per Child EI Hours^a^	
Other	4 (2.9)	All Services	69.00 [56.50,107.50]
Multiple Races	13 (9.4)		
Child Ethnicity^a^		PT	7.00 [.00, 57.00]
Hispanic or Latino	27 (19.4)	OT	.00 [.00, 11.00]
Not Hispanic or Latino	106 (76.3)	ST	28.00 [.00, 56.00]
Unknown	4 (2.9)	DI EI Intensity^a^ (hours per month)	.00 [.00, 15.75] 5.82 [4.67, 8.18]
Respondent Type (mother or female guardian)	132 (95.0)		
Caregiver Education Level			
High school or some college	32 (23.0)		
College degree	40 (28.8)		
Graduate training	67 (48.2)		
Family Income^a^			
$0–50,000	29 (20.9)		
$50,001-100,000	32 (23.1)		
$100,001+	73 (52.5)		

*Abbreviations*: *EI* Early intervention, *PT* Physical therapy, *OT* Occupational therapy, *ST* Speech therapy, *DI* Developmental intervention

^a^Missing data

^b^Respondents could select multiple responses

### YC-PEM e-PRO feasibility (aim 1)

**Fig. 1:**
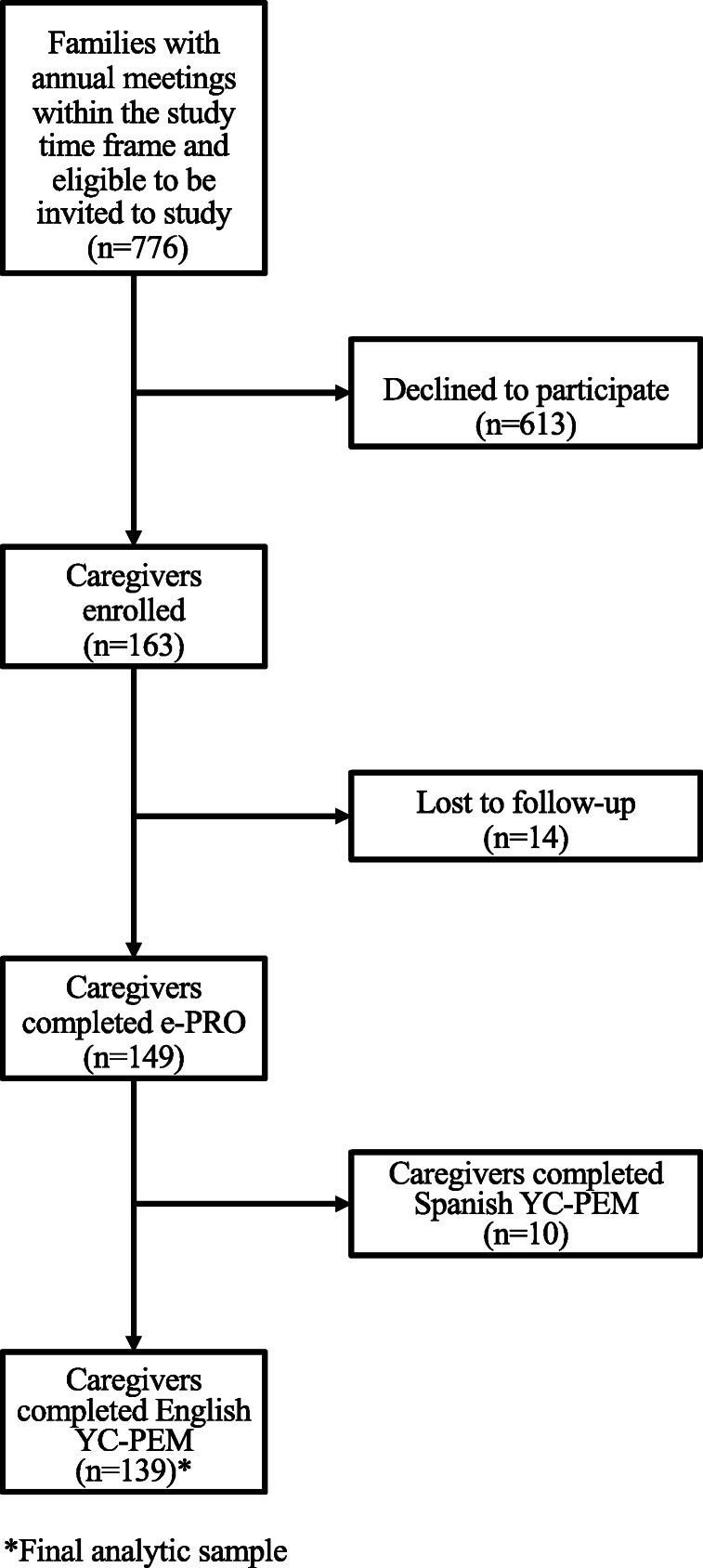
Enrollment diagram

In total, 163 of 776 (21%) families that were eligible to be invited into this study expressed interest, confirmed their eligibility online, and enrolled over 18 months, with the highest enrollment rate (29.2%) occurring following the transition to a standard research protocol in the final 10 months of the data collection period. Fourteen families were either lost to follow-up or declined to participate due to lack of time, lack of interest, or poor timing (i.e. child’s annual review occurred prior to recruitment), resulting in 149 of the 163 enrolled families who completed the YC-PEM e-PRO in English or Spanish. Ten families were excluded based on their completion of the Spanish YC-PEM e-PRO, resulting in 139 families and an 85.3% completion rate (see Fig. 1). Mean completion time was 21.3 min (range = 12.3–29.9). Of those 139 caregivers completing the English YC-PEM e-PRO, approximately four out of every five (80.6%) had no missing data. In cases with missing data, 98.6% of cases had less than 20% of missing data.

### YC-PEM e-PRO acceptability (aim 2)

More than half of the 139 families who completed the YC-PEM e-PRO (58.4%) of the included caregivers perceived the implementation of YC-PEM e-PRO to be at least somewhat helpful. Specifically, 38.0% (*n* = 54) of caregivers perceived the YC-PEM e-PRO as helpful, 20.4% (*n* = 29) as somewhat helpful, and 16.2% (*n* = 23) did not find it helpful. Chi-square results indicated that YC-PEM e-PRO acceptability was not significantly associated with family income categories (χ^2^=.48, *p* = .79) or caregiver education categories (χ^2^= 2.26, *p* = .69).

A subset of caregivers identified supportive and problematic aspects related to the relevance (*n* = 65), wording (*n* = 15), and structure (*n* = 14) of the YC-PEM e-PRO (see Table 2). These caregivers reported that the implementation of YC-PEM e-PRO helped them to reflect on their child’s strengths, challenges and priorities; strategies to promote the child’s participation in current activities; and new activities to try. These caregivers expressed concerns around the relevancy of items based on child age and/or disability; and complex item wording.

**Table 2:** Caregiver feedback on acceptability of the implementation of YC-PEM e-PRO

	Areas of Support	Areas of Concern
Relevance	· Helped me to reflect on my child’s participation, including strengths, challenges, and priorities (*n* = 16) · Helped to think about our helpful strategies and possible new strategies (*n* = 6) · Gave us ideas for activities to participate in (*n* = 5) · It helped by breaking down the day to day activities that are sometimes forgotten about (*n* = 4) · Helped me to think about the impact of the environment (*n* = 3)	· Felt like some questions did not fit our child’s disability and/or young age (*n* = 15) · It did not fit our family situation (e.g., foster parent) and/or focus (e.g., we did not desire any change) (*n* = 10) · Hard to learn from it or relate it to daily life (*n* = 6)
Wording	· Straightforward questions (*n* = 1) · Detailed questions (*n* = 1)	· Wording of some questions was difficult to understand (*n* = 13)
Structure	· Good choice of questions (*n* = 2) · Specificity level of the activities was helpful (*n* = 1) · Questions were thorough (*n* = 1)	· Some questions and/or their response options were overwhelming or difficult to answer (*n* = 10)

*Abbreviation*: *YC-PEM e-PRO* Young Children’s Participation and Environment Measure electronic patient-reported outcome

### YC-PEM e-PRO value (aim 3)

**Fig. 2:**
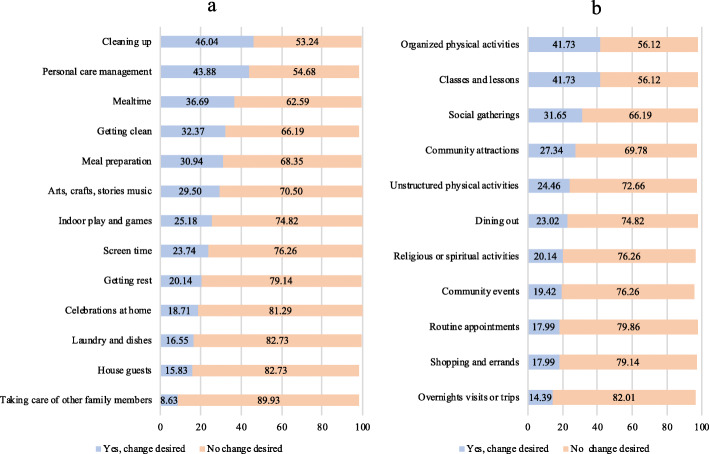
Percent of participation need in home activities (**a**) and community activities (**b**)

Approximately 64% (*n* = 89) of the 139 families who completed the YC-PEM e-PRO proceeded to view their YC-PEM e-PRO report online. On average, caregivers desired for their child’s participation to change in 27% of home activities and 26% of community activities. As shown in Fig. 2, item scores revealed that the most common areas of desired participation change pertained to non-discretionary and structured activities. In the home, 46% of participants desired their child’s participation to change for cleaning up activities, and 44% reported that they desired for their child’s participation to change with respect to personal care management. In the community, 42% of participants reported that they desired for their child’s participation to change in classes and lessons, and organized physical activities.

## Discussion

The YC-PEM e-PRO is a recommended approach to evaluating a young child’s participation in valued activities [19, 20], yet there is critical need to identify supports and barriers to its uptake within a clinical workflow to support its adoption within an EI system of care [23]. This pilot trial contributes additional evidence to the feasibility, acceptability to caregivers, and value when implementing the YC-PEM e-PRO option to support family engagement to plan EI care [22].

### Feasibility

Enrollment rates were similar to prior work, with less than half of the eligible caregivers opting for an electronic option as part of their child’s annual review of progress; however, despite low enrollment, nearly four out of every five caregivers who completed the YC-PEM e-PRO did so in its entirety, an 80% completion rate [22]. Therefore, evidence for the feasibility of implementing the YC-PEM e-PRO in routine EI care was mixed. Feasibility estimates on enrollment in this study were lower than the 50% criterion for feasibility, primarily due to difficulty with provider engagement in recruitment, which resulted in the need to modify the recruitment protocol multiple times during the 18-month data collection period. These problems with recruitment informed a key protocol modification, which is the co-creation of new infrastructure to strengthen EI provider capacity for research engagement [16]. This new infrastructure will support a community engaged research approach to designing and executing a study protocol (inclusive of study subject recruitment) and interpreting and disseminating study results. To our knowledge, there is no precedence for such infrastructure in EI. However, this may be a key driver of successful implementation in future testing, as most EI programs are not university-affiliated and may vary in their research capacity.

Completion time is also similar to prior phases of YC-PEM e-PRO testing [17] and shorter than other family assessment options that are part of usual care (a semi-structured interview that typically takes between 45 min and 2 h to complete). These findings should be interpreted with caution, however, as longer sessions may be needed in some cases to be responsive to family needs and their communication preferences. The YC-PEM e-PRO is not meant to replace usual care but rather to reinforce family-centered care by giving families another option to contribute to an assessment of their child’s needs when planning their child’s EI care [15]. From this perspective, this finding indicates that the online option provides an efficient choice for families to communicate areas of difficulty for their child. Efficiency may be among several factors (e.g., flexibility, privacy) that caregivers consider when choosing to utilize the YC-PEM e-PRO. Thus, our findings suggest that the YC-PEM provides individualized family assessment options that meet the needs of families preferring both short and longer spaces to communicate their family’s needs and priorities for EI care.

### Acceptability

More than half of caregivers perceived the use of YC-PEM e-PRO as at least somewhat helpful. Caregivers often reported that it helped them to reflect on their child’s current participation and their own strategies for supporting it. This was the intended use of YC-PEM e-PRO as shared with EI staff during their initial training, which is to give parents an online way to gather their thoughts about their child, when and where it is most convenient for them to do so. While these results warrant replication with a larger and more diverse sample, these preliminary findings seem to suggest that, for those caregivers who provided such feedback, anticipatory planning may decrease pressure and power differentials [34] that hinder shared decision-making when planning EI care [15].

Some caregivers expressed concern about the relevance of the YC-PEM e-PRO, either because they perceived their child to be too young to participate in activities, or because they perceived their child’s disability to hinder their capability to participate in activities. One potential reason for this finding may relate to parental expectations that their child needs to build developmental skills (e.g., use a pincer grasp) and independently perform functional tasks (e.g., feed self with finger foods) before they can participate in valued activities (e.g., engage in mealtime at home or dine out for meals at a local restaurant). This point of feedback may be best addressed by improving the assessment instructions where the concept of participation is first introduced to parents. This assessment modification should include how participation is distinct from developmental skills and functional capabilities and how it is shaped by child and environmental characteristics [28, 35–39]. Another caregiver concern related to items being difficult to understand, which suggests that wording in the assessment may need modification prior to further testing.

### Value

The intended value of the YC-PEM e-PRO is to help individual families to communicate their needs and priorities, and for the EI program to be able to aggregate these data to learn about common areas of need among the families served and how those needs might change over time as a function of EI service use. The majority of caregivers accessed their online e-PRO report suggesting its value in bolstering family engagement in EI care. However, we acknowledge the lack of data on rates of sharing the YC-PEM e-PRO report with the child’s EI team. Prior studies, though, have indicated caregiver interest in sharing a YC-PEM e-PRO report with service providers [40]. Work is underway to employ a knowledge translation framework for engaging providers and parents as stakeholders to optimize the content and layout of the online report, which may further increase its uptake in an early childhood programmatic context.

Percent desire change estimates were significantly lower in this study as compared to prior phases of study, which could be a function of EI service duration. Whereas children in the prior phase had been enrolled in EI for 7 months on average, families in this study had received services for close to twice that length of time (13.87 months on average). While changes in participation may be gradual, the YC-PEM e-PRO has the potential to detect participation change as a function of EI service use. In an era of accountability, EI programs are pressed to demonstrate high value care [3]. Measures that can detect clinically meaningful change can support these efforts.

### Limitations and strengths

Study results should be interpreted in light of several limitations. The study sample is larger and more diverse than prior phases of testing [41], yet is skewed towards higher education and income levels when compared to the overall enrollment at this EI program [32]. Future studies with more diverse samples according to socioeconomic status may detect greater variability in YC-PEM e-PRO acceptability. These studies may benefit from the use of culturally adapted versions of the e-PRO to maximize reach [21, 42, 43]. Open-ended items to capture caregiver feedback on YC-PEM e-PRO acceptability did not afford for opportunities to clarify and/or further probe about solutions to concerns reported (e.g., allowing parents to provide suggestions for alternative wording) to inform optimizations prior to further testing. Finally, we did not discern between those who were screened ineligible versus those who declined and their reason for declining, resulting in a more conservative enrollment rate. In future phases, EI staff should report on this when recruiting families; similarly, the team should collect demographic or service use data on participants who declined to examine if there are differences between those who enrolled and those who declined.

Despite these limitations, there are a number of key strengths to this study that extend evidence about YC-PEM e-PRO implementation across more diverse EI contexts. This includes evidence of its feasibility when introduced into an EI service coordinator’s routine workflow within a large, non-university affiliated EI program, evidence of its acceptability from the parent perspective, and evidence of its value for providing families with relevant information to view and share with members of their child’s EI team.

## Conclusion

Electronic patient-reported outcomes data may offer a feasible, acceptable and valued alternative for obtaining family input on children’s participation in valued activities to support care planning and outcomes monitoring in EI. Results provide evidence that the implementation of YC-PEM e-PRO may be feasible across EI programs that vary by size and enrollment. Results also extend the evidence of acceptability via caregiver feedback, as well as value by showing the extent to which the e-PRO report was accessed and how the data can be used to detect areas of participation need for individual caregivers and the total EI enrollment. Together, results suggest that the implementation of the YC-PEM e-PRO within an EI clinical workflow merits further study to improve parent activation for shared decision-making.

## Data Availability

NIH funds through the Center for Large Data Research (CLDR) (P2CHD065702; PI: Ottenbacher) were secured to partner with the Inter-university Consortium for Political and Social Research (ICPSR) in curating the data source for public use, effective June 2019 (10.3886/ICPSR37320.v2).

## Abbreviations

e-PRO: Electronic patient-reported outcome
EI: Early intervention
YC-PEM: Young Children’s Participation and Environment Measure
FCC: Family-centered care
fPRC: Family of participation-related constructs
PT: Physical therapy
OT: Occupational therapy
ST: Speech therapy
DI: Developmental intervention
